# Plasticity-Mediated Persistence in New and Changing Environments

**DOI:** 10.1155/2014/416497

**Published:** 2014-10-15

**Authors:** Matthew R. J. Morris

**Affiliations:** Department of Biological Sciences, University of Calgary, 2500 University Drive NW, Calgary, AB, Canada T2N 1N4

## Abstract

Baldwin's synthesis of the Organicist position, first published in 1896 and elaborated in 1902, sought to rescue environmentally induced phenotypes from disrepute by showing their Darwinian significance. Of particular interest to Baldwin was plasticity's mediating role during environmental change or colonization—plastic individuals were more likely to successfully survive and reproduce in new environments than were nonplastic individuals. Once a population of plastic individuals had become established, plasticity could further mediate the future course of evolution. The evidence for plasticity-mediated persistence (PMP) is reviewed here with a particular focus on evolutionary rescue experiments, studies on invasive success, and the role of learning in survival. Many PMP studies are methodologically limited, showing that preexistent plasticity has utility in new environments (soft PMP) rather than directly demonstrating that plasticity is responsible for persistence (hard PMP). An ideal PMP study would be able to demonstrate that (1) plasticity preexisted environmental change, (2) plasticity was fortuitously beneficial in the new environment, (3) plasticity was responsible for individual persistence in the new environment, and (4) plasticity was responsible for population persistence in succeeding generations. Although PMP is not ubiquitous, Baldwin's hypotheses have been largely vindicated in theoretical and empirical studies, but much work remains.

## 1. A Brief History of the Baldwin Effect

Although writers in antiquity recognized that the environment could influence an organism's appearance [1], this did not receive theoretical consideration until Lamarck [2]. For Lamarck, adaptation was the result of the interplay between development and the environment. Phenotypes modified by the environment could then be passed on to offspring. Darwin, in* The Origin of Species* [3], although not denying the possibility of the inheritance of acquired characters [4], focussed on adaptation in a way that downplayed the significance of environmental modifications. With selection acting on chance variations, adaptation became an infinitesimally slow intergenerational process rather than a product of development. The apparent unimportance of environmental modification was confirmed by Johannsen [5] who showed that selection of extreme phenotypes in inbred lines did not result in evolutionary change. By the beginning of the 1900s, Neo-Darwinists and Neo-Lamarckians were divided on the importance of environmental modifications in evolution. However, there were problems with both positions. The Neo-Lamarckians pointed out the difficulty of explaining Neo-Darwinian adaptation in a new environment, since the chance production of fortuitous variation seemed implausible, while the slow pace of adaptation could not match the rapidity of environmental change [6]. On the other hand, no less an authority than Romanes had disavowed most of the evidence for the inheritance of acquired characters [7]. It was into this context that a “not-quite-third” position, that of the Organicists (see Appendix A for glossary), was developed, with a synthesis of positions articulated by Baldwin [6]. The so-called Baldwin effect [8] has proven to be both prescient (Appendix B) and misunderstood (Appendix C). Because of this, it is worth briefly discussing the Organicist position, before highlighting modern evidence for the significance of plasticity in new and changing environments.

Before discussing the Organicist position, it is important to recognize what this position was not: it was not a compromise between Neo-Lamarckism and Neo-Darwinism. It brought Lamarck's first law of use/disuse (and other forms of plasticity) firmly in the camp of Neo-Darwinism, but gutted Neo-Lamarckism in the process (Poulton, quoted in appendix of [6]). In the points that follow, the primary role of natural selection will be readily apparent; Baldwin's organic selection was a supplement to, not a replacement of, natural selection.

**Figure 1:**
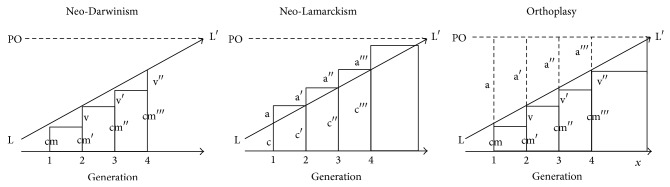
Baldwin's theory of orthoplasy contrasted with Neo-Darwinism and Neo-Lamarckism. Adapted from [6], pp. 187-188. LL′ is the line of evolution. PO is the phenotypic optimum. Primes (′) denote generations. cm is the congenital mean (population mean phenotype, not plastic), v is the genetically based change in population mean phenotype (due to selection on mutations), c is the congenital endowment, and a is the environmental modification of the phenotype. Under Neo-Darwinism, evolution is due solely to the contribution of genetic variation, which is passed from generation to generation; change only occurs through selection on genetic variants. Plasticity may exist, but environmental modifications are not heritable and are therefore of limited adaptive value. Note that evolution in this scenario is directional—mutations that take individuals back to their ancestral phenotype are selected against. Under Neo-Lamarckism, each generation improves its fitness through use and disuse. The initial phenotype is added to by environmental modifications, and this full phenotype is passed on to the next generation as a congenital endowment. As such, the congenital endowment gets closer and closer to the phenotypic optimum with each generation. Finally, under Baldwin's theory of orthoplasy, the first generation has a mean phenotype due in part to heritable variation, but this phenotype is far from the phenotypic optimum. Plasticity adjusts the phenotype to this optimum. These modifications are not passed on from generation to generation, but plasticity itself is; survivors in each generation can thus produce the optimum phenotype in the absence of genetic change. Genetic change does happen, however; any change in the direction of the modified phenotype (and thus the optimum) is favoured, while changes in the opposite direction are selected against. Each generation becomes less plastic, as it falls under greater genetic control. Note that PO is not original to Baldwin's diagram; it was added by the present author for ease of comparison but certainly has its difficulties, particularly regarding costs to plasticity.

The Baldwin effect can be divided into several stages [6]. (1) Organisms are exposed to a novel environment. (2) If all individuals produce unfit phenotypes in the new environment, the population will go extinct. (3) Any organisms that can plastically alter their phenotypes to meet the challenges of the new environment will survive. (4) Natural selection will thus weed out nonplastic or maladaptively plastic individuals, while adaptively plastic individuals will survive and reproduce. (5) The plastic modifications made in the parental generation will not be passed on to their offspring. (6) Plastic individuals will make up a larger proportion of the offspring than in the parental generation. Therefore, many offspring will have the capacity to modify their phenotypes in the same manner as their successful parents. It will look as if they had inherited acquired characters, when in fact they had inherited genetically based plasticity. (7) There is now time for genetic variation to arise to improve the fit between the population and the environment. This can occur in at least one of four ways: (a) coincident variation can occur. Variations arise randomly in the population. Some of these variants may reduce plasticity such that the plastically modified phenotype is negatively affected. These variants will perform poorly and will be weeded out by selection. Other variants will arise that reduce plasticity* in the direction of* the plastic modification. These variants will be favoured and will spread through the population. Thus, although the phenotype itself appears the same from generation to generation, it becomes correspondingly less plastic, until a change in environment will no longer induce a change in phenotype. These coincident variants would be particularly likely to be favoured if there was some cost to plasticity. For instance, learning through imitation could permit offspring to resemble their parents, but the time it takes to learn could be prohibitive, and thus an instinct could evolve through the imitated phenotype becoming genetically assimilated. Steps (1) through 7(a) are generally what is referred to as the Baldwin effect ([8], but see Appendix C), which was Baldwin's primary means of differentiating the Organicists from other perspectives (Figure 1).

Baldwin allowed for other non-mutually exclusive scenarios to play out. 7(b) Variants could arise that increase plasticity, thereby furthering the fit between organism and environment and furthering the extent to which evolution could be directed (as in (a)). (c) Plasticity could, however, increase to the point that genetic change became prohibitive. As a psychologist Baldwin focussed on learning and argued that genetic inheritance could give way to social inheritance brought about by particularly plastic learners. (d) Correlated variants could occur that improve the modified phenotype or other phenotypes permitted to survive by plasticity (in particular, phenotypes that would have contributed to population demise in the absence of plasticity, or phenotypes that could supplant the need for plasticity—see Appendix C for an example of this latter possibility).

Collectively, Baldwin referred to stages (1)–(7) as organic selection, and stage 7(a)–(d) as orthoplasy, or the “directive determination” of evolution through organic selection. For simplicity I will refer to stages (1)–(6) as plasticity-mediated persistence (PMP), while stage 7(a)–(c) involves the numerous possibilities of plasticity evolution (changes to slope and/or elevation of the reaction norm). 7(a) is commonly referred to as genetic assimilation, and 7(a)–(c) as genetic accommodation [9]. 7(d) may, but does not have to, involve genetic accommodation. It is worth pointing out that, for Baldwin, plasticity was largely an adaptation in its own right, a consequence of natural selection, and so maladaptive plasticity and its consequences (such as genetic compensation, [10]) were not envisioned by him.

Now that we have revisited the groundbreaking work of the Organicists, it is time to discuss the evidence for the integral role of PMP in adaptation to novel environments. Evidence for the association between plasticity and persistence will first be reviewed, and then some conceptual and methodological issues that require further attention will be discussed.

## 2. Evidence for Plasticity-Mediated Persistence

**Figure 2:**
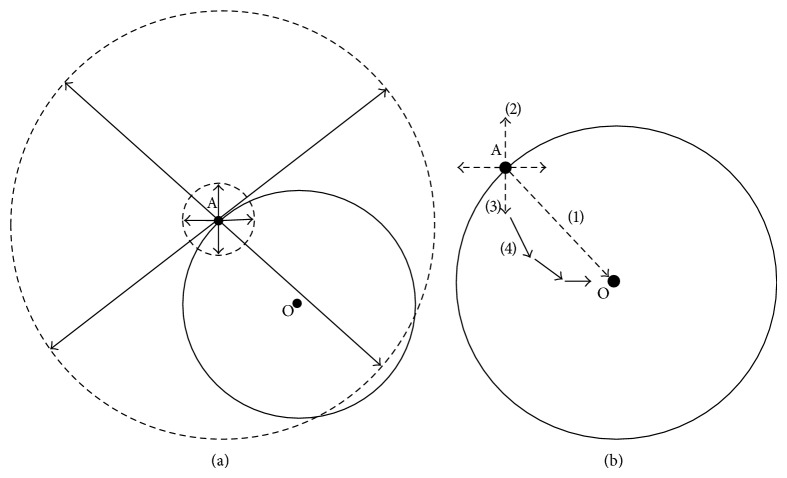
Fisher's geometric model of adaptation used to explain evolutionary rescue and plasticity-mediated population persistence (PMP). In both instances, a population encounters a novel environment with phenotypic optimum O. The distance from the population's mean phenotype (A) to O is a proxy for the strength of selection and therefore for the likelihood of persistence. (a) Evolutionary rescue. The population begins far from the optimum; their phenotypic state in the new environment is shown by A. Mutations of small effect (small arrows, small dashed circle) are just as likely to bring the population closer to the phenotypic optimum (the space denoted by the solid circle) than they are away from the phenotypic optimum. Mutations of large effect (long arrows, large dashed circle) are more likely to move the population away from the optimum than towards the optimum. Upon encountering the novel environment, the probability that a population persists depends on the strength of selection and the likelihood of mutations arising that will move the population towards the optimum. Similarly, standing genetic variation could move the population to its optimum rapidly, with the rare mutations of large effect that had persisted in the old environment suddenly being favoured, or numerous small effect variants shifting in frequency (as per [128]). (b) Plasticity-mediated persistence (PMP). In this case A represents the phenotype in the old environment, and the dashed arrows represent how the phenotype can change upon exposure to a new environment. (1) Plasticity is perfect and the population is under stabilizing selection. (2) Plasticity moves the population away from the optimum, reducing its likelihood of persistence. (3) Plasticity is imperfect but moves the population towards its optimum. (4) Imperfect plasticity permits the population to survive long enough for evolutionary rescue (solid arrows) to occur. (2) and (3) combined could represent the random nature of plasticity revealed in the new environment through the uncovering of cryptic genetic variation. Figures adapted from [97, 129, 130].

The first step of the Baldwin effect involves the persistence of individuals and populations in novel environments, with such persistence attributable to phenotypic plasticity (Figure 2). PMP is satisfied under the following conditions. (1) No evolution: all individuals produce optimal plastic responses in the new environment. (2) Persistence awaiting evolutionary rescue: all individuals produce suboptimal plastic responses in the new environment that permit persistence long enough for more fit genotypes to arise and spread. (3) Selection on standing genetic variation: some individuals are adaptively plastic and are favoured in the new environment. PMP does not occur if plasticity evolves from* de novo* mutations after colonization. A wealth of empirical and theoretical evidence has accumulated that suggests or directly demonstrates PMP in natural populations, particularly from studies on evolutionary rescue during climate change, work on invasive species and colonization success, and a disparate group of experiments on natural populations.

### 2.1. Evolutionary Rescue and Climate Change

When populations encounter novel environments through dispersal or climate change, genotypes that were fit in the old environment may no longer be fit in the new environment (Figure 2). The time it takes for more fit genotypes to arise may be prohibitively long, resulting in population extinction. Yet, successful colonization via adaptive evolution does occur, a scenario known as evolutionary rescue (ER) [11, 12]. ER can take the form of* de novo* mutations [10], standing genetic variation [13], or the introduction of adaptive alleles from migrants [14]. The interaction between ER and plasticity has only recently been addressed [11].

ER may not be possible in coarse-grained environments [15]. Plasticity, by slowing the rate of population decline, can overcome this hurdle, providing time for beneficial mutations to arise [16]. This has been confirmed empirically: the likelihood of extinction for great tits increased 500-fold in the absence of plastic responses to climate change [17]. This was largely true in thirteen other bird species, although faster generation times offset the need for plasticity [17]. Climate change has resulted in population declines of numerous nonplastic species, suggesting that ER does not have time to work in the absence of plasticity [18, 19]. For instance, rising temperatures shifted flowering time but not West Greenland caribou calving time, producing a trophic mismatch that declined calf production fourfold [20]. Species that can plastically maintain synchrony with peak food production have shown no such population decline [21, 22]. However, there is no consistent rule. Costs and limits of plasticity can constrain plastic responses, resulting in higher fitness for populations that do not plastically improve synchrony [23, 24], while ER can operate to improve synchrony in the absence of plasticity [25]. The above evidence therefore supports the prediction that plasticity is sufficient but not necessary to promote persistence, and that ER can occur in the absence of plasticity.

### 2.2. Invasive Success

Invasion is a three-step process: dispersal, establishment, and range expansion [26]. Factors besides plasticity have been identified in invasive success (e.g., [27–29]), but plasticity may also play a role.

#### 2.2.1. Dispersal

In order to successfully invade a new environment, individuals must first disperse to that environment. PMP predicts that species with high dispersal rates should also be highly plastic, as dispersal involves encountering spatial heterogeneity [30]. Indeed, dispersal of nonplastic organisms can reduce the likelihood of successful colonization by introducing maladaptive alleles to colonizing populations [31]. Hollander [32] tested the relationship between dispersal and plasticity in 258 species of marine invertebrates and found that on average dispersing species were more plastic than nondispersing species. Presumably, without such plasticity dispersers would fail to colonize the locations to which they disperse. Modelling work has shown that the relationship between the period of environmental sensitivity and the timing of dispersal matters: if plastic modifications to the organism occur irreversibly before dispersal, colonization success is low; if it occurs after dispersal, colonization success is much higher [33]. However, it is clear that many species have high dispersal rates and low levels of plasticity, so plasticity is again sufficient but not necessary for colonization [32]. Rapid generation times, for instance, may allow colonizing populations to rapidly evolve to meet the demands of the new environment [17].

Dispersal itself can be plastic in a manner that inhibits or improves invasive success. Traits associated with dispersal can be plastic in a way that maximizes dispersal within a habitat but limits the possibility of encountering new habitats [34]. On the other hand, dispersal can increase under stress, exposing offspring to novel environments [35]. Invasive species may therefore exhibit higher dispersal immediately upon colonization of stressful environments, simultaneously expanding their range [26] while decreasing their probability of establishment through a reduction in population size [33]. So plasticity in dispersal rate may inhibit persistence.

#### 2.2.2. Establishment and Range Expansion

Invasive species often display a suite of functionally relevant plastic modifications upon encountering a new environment (e.g., [36–38]), but it is not always clear if this plasticity is necessary for persistence or if, in the absence of plasticity, invasive success would still be high. For instance, Lande [39] showed that preexistent plasticity could rapidly evolve during colonization. This model received empirical support through the repeated evolution of increased plasticity for ion-motive enzyme activity during multiple colonization events of freshwater by a marine copepod, likely through repeated selection on preexisting standing genetic variation [40]. But this only shows that plasticity was beneficial, not that it was necessary for colonization. A model by Thibert-Plante and Hendry [33] deserves empirical support, as it demonstrates a 40% colonization success rate for nonplastic individuals but an 87% colonization success rate for plastic individuals, with the importance of plasticity increasing with the strength of selection. This suggests that plasticity is at least sometimes required for establishment.

The PMP hypothesis has led to predictions about the degree of plasticity that should be evident in invasive species [41]: (1) invasive species should be plastic for relevant adaptive traits; (2) invasive species should be as or more plastic than their ancestors; (3) invasive species should be more plastic than the species they displace; (4) plasticity should be greater in invasive species than in noninvasive species. The last year alone has seen numerous studies testing these predictions (Table 1), while several reviews discuss the current state of the matter (Table 2). A related prediction, that plastic species should have greater geographic ranges, has received limited empirical support [42–45].

The most common research program simply measures plasticity in invasive species and concludes that such plasticity may have contributed to invasive success. Although interesting, this tells us little about PMP. It is usually unknown if plasticity evolved in the new environment or preexisted prior to invasion. Comparison between populations of invasive species from their native and introduced ranges helps address this question—if plasticity is similarly present in both forms, it suggests the preadaptive significance of plasticity [46], while if plasticity is higher in the invading populations it suggests that plasticity was imperfect but adaptive [39]. Resurrection studies, where seeds from plants collected during the initial phase of invasion are grown with seeds collected generations later, have confirmed that plasticity often increases during invasion [47, 48]. Such studies are still not ideal, as they do not address whether plasticity itself was essential for invasive success.

One way to address PMP is to compare plasticity between invasive species and their native competitors. If plasticity is essential for colonization, theory suggests that invasive species will be more plastic than the species they displace. If plasticity is unessential, there will be no such trend. Many individual comparisons have been conducted with equivocal results. Meta-analyses, however, support PMP (Table 2): although there is variation among comparisons, invasive species are on average more plastic than their competitors [49, 50]. Such comparisons, however, suffer from interpretive problems. Invasive species may only need to be* as *plastic as their competitors in order to meet the challenges of a new environment (“join-the-local” hypothesis [51]). More alarmingly, these studies are usually performed under isolation and for contrived phenotypes and environments [52], but the extent and significance of plasticity for invasive success can only be assessed under natural conditions with competition. An ideal experiment was conducted by Engel and Tollrian [53]. Invasive and native species of* Daphnia* were raised in the presence and absence of predators, separately and while competing. The nonplastic native species outperformed the plastic invader while competing in the absence of predators, but morphological plasticity in the invader gave the invader a competitive edge under predation. In this case clear predictions about the significance of plasticity could be made and assessed; for many phenotypes measured in invasive species the significance of plasticity is less clear.

A second way to establish PMP is to compare plasticity between invasive species and noninvasive conspecifics that have been introduced to but did not become established in new environments. (Note that some studies compare invasive species and “noninvasive” species, where the noninvasive species is simply the species being displaced by the invaders. These studies address prediction number 3 above and are prone to its limitations (see, e.g., [54]).) Invasive birds and mammals tend to have greater plasticity in learning than their noninvasive conspecifics (Appendix D). One particularly interesting study compared invasive and noninvasive populations of the same species. In the plant* Centaurea stoebe*, both diploid and tetraploid populations occur across its natural European range, but in North America tetraploids have been the only successful invaders. This is despite numerous opportunities for diploid establishment. A comparison in plasticity of physiological and life-history traits in all three groups showed greater plasticity in tetraploids, but no difference in plasticity between American and European tetraploids. The direction of plasticity appeared to be adaptive under North American climatic conditions, suggesting plasticity's important role in invasive success in this species [55].

#### 2.2.3. Plasticity in Response to Invaders

Introduced species provide a new environment to which native species can respond. Plastic responses in the native species may facilitate persistence in the face of such introductions. For instance, behavioural plasticity upon exposure to an introduced predator may prevent the collapse of the prey population [36, 56, 57]. Plasticity in the native species may even prevent successful invasion of the introduced species [58]. On the other hand, inappropriate plastic responses to invaders may inhibit persistence [59].

### 2.3. Other Evidence

Studies from common garden experiments have provided some evidence for PMP. For instance, genotypes of* Impatiens capensis* that inhabit distinct environments and which differ in their degree of plasticity were transplanted into the respective genotype's habitat. The plastic genotype performed better in the nonplastic genotype's environment than did the nonplastic genotype in the plastic genotype's environment, and this was associated with the magnitude of phenotypic convergence between native and transplanted genotypes [60].

Knowledge of the ancestor-descendent relationship between populations has provided some of the most striking evidence for PMP. Yeh and Price [61] studied a population of dark-eyed juncos on the campus of the University of California, San Diego, invaders that had left a montane population and successfully colonized this new environment in the 1980s. A six-year study beginning in 1999 found that the warmer coastal environment permitted a longer breeding season, and more offspring per breeding pair, than the montane climate. Despite this increased reproductive output, the population remained stable with little migration. They concluded that, in the absence of plasticity, the population would not have survived, presumably due to higher rates of predation on campus than in the wild [61]. Benthic and limnetic threespine stickleback are relatively nonplastic in the morphological features that distinguish them, but they occupy opposite ends of the morphological reaction norm of ancestral marine stickleback, suggesting that ancestral plasticity was required for these species pairs to occupy their respective niches (the “flexible-stem” hypothesis; [62, 63]; see also [64–75]).

Suggestive evidence for PMP comes from other sources. Phenotypic change is greater in populations experiencing anthropogenic disturbance than in those that are not, and this primarily occurs through plastic responses that may be adaptive [76, 77]. Studies of tolerance limits have shown that many species are not living at the edge of what they can tolerate, suggesting that physiological and molecular plasticity will permit persistence under future climate change [78, 79]. Finally, phylogenetic analyses have allowed intriguing correlational questions to be asked. Pfennig and McGee [43] hypothesized that, if PMP is true, clades with plasticity should be more speciose than sister clades of similar age that lack plasticity, due to a combination of reduced extinction risk, greater opportunities to diversify, and, unrelated to PMP, increased evolvability of plastic traits. Their predictions were supported in three fish and two amphibian lineages. Overall, from climate change to invasive species to phylogenetic approaches, plasticity does seem to be an important contributor to persistence in new or changing environments.

## 3. Criteria for Demonstrating PMP

Although the above examples demonstrate that plasticity is an important means by which species cope with changing environments, many of these examples only infer PMP without directly demonstrating it. This is in part because the definition of “mediated” in PMP is open to debate. Baldwin envisioned a situation in which nonplastic individuals were less fit than adaptively plastic individuals. If no individuals were plastic, the population as a whole would go extinct, barring (we would add today) evolutionary rescue. This can be called hard PMP and is empirically demonstrated only when plasticity is shown to be responsible for the survival of the individual and/or production of offspring in the new environment. Hard PMP can be contrasted with less ambitious but no less interesting research programs that infer that preexistent plasticity has some adaptive significance in a novel environment, what can be called soft PMP. Soft PMP studies usually cannot rule out alternatives to PMP; for instance, an organism may fortuitously produce an adaptive phenotype in a new environment, but it may have been able to survive and produce offspring even in the absence of this plastic modification. In the absence of evidence for hard PMP, a consilience of evidence from soft PMP studies is required to stack the evidence in favour of PMP. Ideally any study seeking to demonstrate hard PMP will be able to address the following questions.

### 3.1. Q1: Does Plasticity Preexist Environmental Change?

By definition, PMP can only occur if plasticity is retained within the population in the ancestral condition—that is, the potential for plastic modification must preexist the new environment. Many plasticity studies that indirectly infer PMP are not geared to address this question, such as comparisons between plasticity in invasive versus noninvasive species. In such cases the evolution of plasticity after invasion cannot be ruled out—perhaps invasive species have genomic architectures that are more prone to plasticity evolution [80]. In order to demonstrate PMP, there must be some way to measure plasticity immediately upon exposure to a novel environment, or to infer preexistent plasticity from ancestor-derived comparisons or phylogenetic analyses.

Experimental environmental manipulation and studies on natural populations during environmental change have shown that plasticity can indeed preexist environmental change. Some particularly fascinating examples involve the production of novel phenotypes under unnatural environments. Moss grown under zero-gravity conditions developed unusual spiral morphologies [81]; a killer whale at Marine Land learned to catch gulls by baiting them with its fish food, a game it then taught to others [82]; the recent northern range expansion of a butterfly species resulted in the production of a novel wing colouration induced by temperature [83]; experiments under unnaturally high carbon dioxide levels altered flowering time and carbon stores in numerous plant species (e.g., [84, 85]); invasive black rat populations developed a novel strategy for accessing pine seeds [86]. Studies on developmental plasticity have shown that tissues can respond in remarkable ways to environmental input, resulting in dramatic morphological changes within an individual. The so-called “two-legged goat effect” was named by West-Eberhard [9] after a goat that was forced to walk on two legs due to a congenital limb defect. This resulted in the development of a “kangaroo-like” skeletal system. Similar observations were made even earlier by Fuld on a “kangaroo-like” two-legged dog [87]. Most recently this effect was observed in the extant stem tetrapod* Polypterus*, a fish that can breathe and “walk” on land. When raised in a terrestrial environment, developmental plasticity resulted in morphological changes that improved terrestrial performance and pointed towards a preexistent plasticity explanation for the origins of some aspects of the terrestrial body plan [88].

Comparisons between contemporary ancestral and derived populations may also be used to infer preexistent plasticity. If both forms are similarly plastic, it is tempting to infer that plasticity permitted persistence in the novel environment. However, caution should be exercised: plasticity in the ancestral form must be tested in the derived form's environment [89]. If plasticity is measured in the ancestral form in the ancestral environment, it is possible that, under derived conditions, this plasticity would be hindered, necessitating its reevolution [10]. If the derived form is not plastic but the ancestor is, care should be taken to demonstrate that canalization occurred in the derived form, and not that plasticity subsequently evolved in the contemporary “ancestral” form. This is straightforward to do if multiple derived forms exist that have nonplastic phenotypes representative of opposite ends of the ancestral reaction norm. This is the basis of the “flexible-stem” hypothesis discussed above (e.g., [62]).

### 3.2. Q2: Is Preexistent Plasticity of Utility in the New Environment? 

Simply demonstrating preexistent plasticity is not enough, as this plasticity may be maladaptive, adaptive, or neutral in the new environment. Some sort of utility in the new environment needs to be demonstrated. A novel colour phenotype induced by rising temperatures is interesting, but does it have fitness consequences? Others have reviewed the steps taken to demonstrate adaptive plasticity in nature [90], but not all of these steps apply to preexistent plasticity. For instance, associating the degree of plasticity with the extent of environmental heterogeneity will not work if plasticity is retained in ancestral organisms that do not experience the inducing environment. Strong evidence for preexistent plasticity's adaptive significance comes from flexible-stem scenarios (e.g., [62]). Barring ancestor-derived comparisons, selection experiments on individuals that differ in their degree of plasticity, under novel environmental conditions, would be ideal, but studies that logically consider the function of the modified phenotype in the novel environment would suffice. For instance, learned behaviours that exploit novel food resources do not require further justification to show that they have significance within the new environment. No matter the evidence, it is essential that the preexistence of plasticity and the significance of this plasticity in a new environment be demonstrated together; on their own neither is sufficient to demonstrate PMP. Unfortunately, most studies cited as demonstrating PMP stop here [75] and do not actually demonstrate that plasticity itself is essential for persistence.

### 3.3. Q3: Is Plasticity Responsible for Persistence of the Individual in the New Environment? 

This is the question that soft PMP studies do not directly demonstrate. Important modeling work and meta-analyses suggest that under some conditions at least individuals would perish without plasticity ([16, 32, 33]; however note that individual and population persistence are generally not separated), but direct empirical demonstrations are limited. For instance, Yeh and Price [61] discovered presumably preexistent plasticity that was of utility in the new environment and inferred its role in persistence by demonstrating that, despite the plastic increase in reproductive output, the population remained relatively stable. They associated this stability, rather than population growth, with increased cat predation and from there concluded that, in the absence of this reproductive plasticity, the population would decline. The logic is sound, but the conclusion is inferred. Empirical studies explicitly linking plasticity with individual persistence are sorely needed.

The nature of the question reveals two different ways of thinking about “mediated” that were not captured by the hard versus soft PMP distinction already made. “Mediated” could be relative to other genotypes in the population. Under competition in a new environment, those individuals with preexistent plasticity may outperform nonplastic individuals, resulting in an increase in the frequency of plastic individuals within the population (e.g., [53]). Nonplastic individuals perish; plastic individuals thrive. However, this tells us little about what would have happened had preexistent plasticity not existed. Would the nonplastic genotypes have fared so poorly in the new environment in the absence of competition? “Mediated” then could also refer to situations in which nonplastic individuals perish in the absence of competition with plastic genotypes. Plasticity does not just give an edge over competitors—it is essential for persistence in the new environment. Baldwin was not explicit in differentiating between these two forms, but current researchers should be. The most obvious way to address this question is to measure survival of plastic and nonplastic phenotypes under novel environmental conditions, together and separately (e.g., [53]).

### 3.4. Q4: Is Plasticity Responsible for Persistence of the Population in the New Environment?

This question is really an extension of the question above. If plasticity is favoured by selection from a pool of individuals that differ in plasticity, or if without plasticity the individual would perish, it stands to reason that such plasticity would also be responsible for the continued survival of the population in the new environment. These questions need to be uncoupled for three reasons. First, individual persistence is an intragenerational process, while population persistence is an intergenerational process. Donnelly et al. [91] conclude a lengthy review on plasticity and rapid adaptation to climate change in trees, insects, and birds, by suggesting that plasticity may have immediate fitness-improving effects, but over longer time periods evolutionary change will be required for populations to persist. This is in line with Baldwin's hypothesis that plasticity could permit survival long enough for necessary genetic changes to occur. This highlights a crucial point in the definition of PMP: for PMP to be satisfied, it does not require that the population survive indefinitely due to plasticity. So long as plasticity can keep enough individuals around for ER to occur, PMP has occurred.

Second, and in association with this, persistence is a sneaky term that researchers may define in different ways. Measures of survival, for instance, will say little about fecundity; but fecundity is required if the population is to persist. Third, individuals and populations survive under different temporal scales and therefore may experience different levels of environmental heterogeneity. Plasticity in some traits may permit individuals to survive in the short term but be insufficient to meet the challenges of longer-term environmental change. Modeling work strongly supports the conclusion that plasticity can be essential for colonization success or population persistence under changing environments ([16, 17]; see evolutionary rescue discussion above), but experimental work in which the long-term colonization and establishment success of plastic and nonplastic genotypes are compared is needed.

### 3.5. Conclusion

Soft PMP studies tend to infer yes to questions three and four based on empirical affirmations to questions one and two (e.g., [75]). But questions one and two are not sufficient to empirically demonstrate PMP. For instance, surface-dwelling progenitors of cavefish harbour cryptic genetic variation for eye size. Under cave environments, the heat shock protein 90 system is unable to maintain proper function, resulting in the phenotypic expression of this genetic variation. Selection then favours variants in the direction of reduced eye size [92]. Although this study shows that adaptive plasticity leads to the development of smaller eyes in cave-dwelling fish, it cannot rule out the possibility that, in the absence of such plasticity, vestigial eyes would have still evolved. Ideally, hard PMP studies will become more common in the future, by demonstrating that plasticity is preexistent, functionally significant, and directly responsible for the persistence of the individual and the population. Such studies naturally produce tangential questions that are not necessary for demonstrating PMP, but which are interesting in their own right.

## 4. Additional Questions Raised by PMP

### 4.1. How Is Preexistent Plasticity Maintained in the Population?

Why would plasticity exist before the new environment was encountered? There are numerous possibilities, the first of which has received the least amount of attention. (a) In the case of the colonizing environment having the same environmental fluctuations as the old environment, plasticity may have evolved as an adaptation to the old environment and maintained its adaptive function in the colonizing environment. This tends to be underappreciated because it seems obvious, but often novel environments vary in other factors that may negatively interact with the reaction norm of interest. If a phenotype adaptively responds to temperature in the old environment, the new environment may have the same temperature fluctuations but a higher salinity which inhibits the expression of the temperature-induced reaction norm. Researchers tend to ignore phenotypic similarity between populations, but this is a fruitful avenue for further research, as it could uncover examples of genetic or plastic compensation [10, 93, 94]. (b) Ancestral plasticity may have evolved in the old environment and be sufficient to partially meet the challenges of the new environment, favouring ER [16, 39]. (c) Plasticity may have existed in the ancestral population without being expressed.

Of particular theoretical interest for (c) are instances in which populations live below their tolerance limits. As one example, sea urchins exposed to low pH increased transcript abundance for genes associated with biomineralization. This transcriptional plasticity restored the morphological phenotype, yet was responding to acidity levels never experienced in nature [79]. Physiological stress responses may be capable of compensating for these rarely-if-ever encountered environments [94]. Unexpressed plasticity may also be caused by the inherent capacity of organismal structure and behaviour to change in response to novel stimuli in a way that permits proper organismal functioning in the novel environment. This is the “two-legged goat effect” described above [9, 88]. Finally, unexpressed plasticity may be caused by cryptic genetic variation (CGV). CGV is defined as standing genetic variation that has no impact on phenotype or fitness under normal conditions, but which is expressed under stressful or novel conditions [95, 96]. CGV can be accumulated due to neutral mutations that occur in unexposed regions of a reaction norm [97], past selection in other environments [98], or the action of phenotypic capacitors like Hsp90 that reduce the phenotypic effects of novel genetic input [92, 99]. Novel conditions express these hidden variants, resulting in increased genetic variance for the trait. The random nature of this revealed plasticity may result in some variants being more fit than others and a subset of CGV being selected. Experimental evidence supports the importance of CGV for PMP and rapid adaptation ([40, 100, 101]; reviewed in [75]). Importantly, the very nature of plasticity enables the accumulation of CGV in two ways [63, 102]. (a) Since plastic phenotypes are only expressed under particular circumstances, if conditions to produce the phenotype are not met, mutations can accumulate in the unexpressed regions of the reaction norm and be revealed in the new environment. (b) The development of plastic phenotypes may require more genes than nonplastic phenotypes. This provides a larger number of nucleotide sites for mutations that have conditional expression [103].

### 4.2. Under What Circumstances Will PMP Occur?

This is perhaps the biggest and most challenging question facing future work on PMP. Genetic compensation occurs when maladaptive plasticity is overcome through evolutionary rescue. That is, there are situations in which a nonplastic genotype is more fit than a plastic genotype, and yet these maladaptively plastic individuals survive, reproduce, and eventually reevolve their old phenotype through a new developmental pathway [10]. Under what situations, then, is adaptive plasticity so essential that without it the individual would perish and the population would go extinct? Just how common is PMP? Is it possible that under most scenarios plasticity is merely beneficial without being necessary for survival?

### 4.3. What Types of Phenotypes Are Responsible for PMP? 

It is likely that many of the phenotypes essential for persistence are transitory and/or molecular or physiological and are therefore difficult to measure [104]. Researchers may not even be aware that plasticity is occurring—ancestral and derived populations may be, at a whole-organism level, phenotypically similar, while plasticity occurs behind the scenes [93, 94]. That is, phenotypic similarity may be caused by different developmental networks induced by different environments, and without this plastic compensation, extinction may result. Empirical demonstrations of PMP may become increasingly more common as these molecular phenotypes become more readily accessible.

### 4.4. Is Variation in Plasticity due to Genetic Variation? 

Variation in plasticity may not always be due to genetic variation. An individual's degree of plasticity may have to do with the amount of environmental heterogeneity experienced early in life [105]. A consistently expressed environment may cause the expression of a plastic phenotype early in life, followed by an insensitivity to further environmental change, while the experience of random heterogeneity may increase the duration of environmental sensitivity. Thus genetically identical individuals of the same age class may have very different phenotypic responses to a novel environment. Those individuals that persist, and that contribute to the persistence of the population, may persist for reasons other than genetic variation. Furthermore, the role of cross-generational epigenetic inheritance in population persistence is only now being elucidated [75]. For these reasons, unless genotypes are directly measured, one should be cautious about calling individuals with different plasticities different “genotypes.”

### 4.5. Can PMP Be Linked with Ecological Speciation?

There are three components of ecological speciation: the colonization of distinct environments; adaptive phenotypic and genetic divergence under divergent selection; and reproductive isolation as a byproduct of divergent selection, with reinforcement as a potential additional step [106]. Plasticity can play an important role in each of these steps of speciation (Table 3), with the most obvious step involving survival and phenotypic divergence in divergent environments. However, preexistent plasticity has the ironic role of prohibiting the next stage of ecological speciation, genetic divergence, by permitting repeated and successful migrations between phenotypically divergent populations [33]. The direct connection between PMP and ecological speciation remains intriguing but an as yet unverified possibility.

## 5. Conclusion

Over one hundred years ago, Baldwin predicted that plasticity could permit species to survive in changing environments and that plasticity could favour both coincident and correlated variants. He developed these ideas primarily to rescue plasticity from Lamarckian inheritance. In 1953 Simpson [8] concluded that, although intuitive and in line with current evolutionary theory, there was no compelling evidence for the Baldwin effect in nature. Today that story has changed. A consilience of evidence from soft PMP literature is highly suggestive of plasticity's integral role in persistence. Hard PMP can be demonstrated only if preexistent fortuitously beneficial plasticity is measured and directly linked with individual and population persistence. Theoretical and empirical research has proven promising, but much work remains to be done.

## Figures and Tables

**Table 1:** Five important questions pertaining to PMP and invasive success, and references pertaining to these questions published in 2013.

Organism	Description	Reference
**Question 1: Are invasive species plastic for functional traits?**		
Cyanobacteria	Growth rate and morphology were altered by temperature.	[131]
Plant	Physiological plasticity permitted savannah-adapted trees to survive floodwaters throughout their invaded range.	[132]
Plant	Climate variation induced plasticity in several phenotypes.	[133]
Plant	Different growth strategies in different habitats kept population growth stable.	[134]
Plant	Reciprocal transplant of 15 invasive populations showed that all populations were similarly plastic.	[135]
Plant	Plasticity was induced by water depth and light quality.	[136]
Plant	Different populations of invasive species differed in plasticity to changing water conditions.	[137]
Plant	Growth was altered by nitrogen concentrations.	[138]
Mollusc	Size-at-maturity changed with temperature, permitted survival during El Niño.	[139]
Mollusc	Shell shape plasticity induced by water flow velocity.	[140]
Crustacean	Reproductive plasticity detected as facultative parthenogenesis.	[141]
Insect	Acclimation to cool temperatures increased performance.	[142]
Insect	Physiological plasticity enabled salt tolerance in invaded island habitats.	[143]
Fish	Plasticity found in length of spawning season.	[144]
Amphibian	Hydroperiod did not affect growth or development (no plasticity detected).	[145]
Bird	Epigenetic modifications higher in populations with less genetic diversity.	[146]

**Question 2: Are invasive species as or more invasive than their ancestors?**		
Plant	Invasive populations more plastic than populations from the ancestral range.	[147]
Plant	8 invasive populations were as plastic as 8 populations from the ancestral range for 20 highly plastic traits.	[148]
Plant	2 invasive populations had evolved increased and decreased plasticity for different traits, in comparison to 18 populations from the ancestral range.	[149]
Plant	Plasticity increased in the invasive population relative to their resurrected ancestors.	[48]
Fish	Invasive populations were less plastic than populations from their ancestral range.	[150]

**Question 3: Is plasticity higher in invasive species than in the competitors they are displacing?**		
Plant	Germination of invasive species was not affected by salinity, presumably implying physiological plasticity; one native species performed even better.	[151]
Plant	Invasive species were more plastic than native species and were better competitors, but this varied with the invasive success of the species.	[152]
Nematode	Plasticity in the reproductive traits of an invasive species gave it a competitive advantage.	[153]
Insect	Physiological plasticity to temperature was higher in an invasive species.	[154]

**Question 4: Is plasticity higher in invasive species than in noninvasive species?**		
Insect	An invasive species with a large range was compared to an invasive species with a small range on the same island; the large-range species was more resistant to temperature, implying physiological plasticity.	[45]

**Question 5: Does plasticity permit persistence of native species in the face of invaders? (native/invader)**		
Crustacean/ Crustacean	Invaders were only present in ion-rich waters, natives in ion-poor and ion-rich waters. Plasticity in natives allowed ion-poor populations to migrate to ion-rich waters, supplementing a dwindling ion-rich native population.	[155]
Insect/Insect	Parasitoid wasps ably preyed upon an invading moth, irrespective of moth's host plant.	[156]
Amphibian/Insect, Fish, Crustacean	Both native and invasive amphibians exhibited behavioural and/or morphological plasticity in the face of both native and invasive predators, although the magnitude of the plastic response was smaller towards invasive predators.	[157]

**Table 2:** Important review papers, meta-analyses, or large-scale experiments on plasticity and invasive success.

Species type	Number of species	Overall results	Reference
Plants	79 native-invasive species comparisons	Trend for greater plasticity in invaders but better performance in natives; plasticity favoured better performance in disturbed environments.	[49]

Plants	5 native-invasive species comparisons	Trend of higher plasticity for invaders, under resource limitation.	[50]

Plants	10 invasive-ancestral population comparisons	In 6 of 10 cases, invaders were more plastic than their progenitors.	[47]

Plants	7 native-invasive species comparisons	Species relatedness was a better predictor of plasticity than invasiveness.	[158]

Plants	75 invasive-native or invasive-noninvasive species comparisons	Invasive species were on average more plastic, but this was not always associated with a fitness benefit.	[54]

Plants	35 invasive-native or invasive-noninvasive conspecific species pairs	Invasive species were, on average, as plastic as their conspecifics.	[159]

Plants	211 species with different levels of invasiveness	The most widespread invasive species were also the most plastic, increasing biomass with resource abundance.	[44]

Plants	12 invasive and 12 native species in shrub community	Invaders on average displayed both robustness to poor environments and increased plasticity under favourable environments.	[160]

Plants	330 invasive and 959 native flowering plants	On average invaders had shifted their flowering time with climate change while natives had not.	[161]

Insects	2 invasive and 4 native species	No difference in the extent of plasticity, but natives performed better under cool acclimation and invaders performed better under warm acclimation.	[162]

Vertebrates	???	An extensive review on the ways species have coped with urban environments, including behavioural plasticity.	[77]

Birds	39 successfully and unsuccessfully introduced species	Larger relative brain size associated with invasive success.	[118]

Birds	69 species, 501 introduction attempts	Larger relative brain size associated with invasive success.	[119]

Birds	196 species, 646 introduction attempts	Larger relative brain size associated with increased innovation and invasive success.	[120]

Birds	202 species, 832 introduction attempts	Larger relative brain size and broader ecological niches associated with invasive success.	[122]

Mammals	100 species, 513 introduction attempts	Relative brain size important predictor of invasive success.	[121]

**Table 3:** Ways in which plasticity may facilitate or hamper ecological speciation.

Process of ecological speciation	Plasticity facilitates speciation	Plasticity hinders speciation
Colonizing divergent environments	PMP (i) Preadapted plasticity (ii) Cryptic genetic variation	Maladaptive plasticity

Divergent selection on divergent phenotypes in divergent environments	Production of divergent phenotypes (i) Adaptive plasticity (ii) Innovation/novelty (iii) Phenotypic accommodation Production of genetic variation (i) Mutations in conditionally expressed genes (ii) More targets for mutation Coincident variation: genetic assimilation (i) Modified phenotype is adaptive (ii) Plasticity then lost via neutral mutations or selection to reduce costs (modern definition) Correlated variation (i) Enhance the modified trait (ii) Improvement of traits “saved” by plasticity (iii) Novel selection imposed by changes to phenotypic correlations	Migration and postdispersal plasticity erode genetic differentiation Divergent phenotypes in divergent environments, generated by plasticity, weaken selection in the new environment

Reproductive isolation as a byproduct of selection	Plastic changes isolate populations if plasticity is irreversible and assortative mating occurs Genetic assimilation/correlated variation leads to isolation (e.g., reduced hybrid fitness)	Migration and postdispersal plasticity erode genetic differentiation Plasticity in sexually selected traits removes reproductive barriers (hybrid swarms)

## Appendices

### A. Glossary of Terms

*Baldwin Effect*. The process whereby plasticity facilitates survival in a new or changed environment and directs the future course of evolution by favouring coincident and correlated variants. Possible outcomes include the evolution of greater plasticity, enhancements to the modified trait, selection on other traits, or genetic assimilation.

*Canalization*. The developmental stabilization of a phenotype against environmental or genetic perturbations. Canalization could occur for multiple or individual phenotypes along a reaction norm.

*Coarse-Grained Environment*. A term used to refer to environmental change that occurs between generations, either temporally or, for dispersing organisms, spatially. Contrasted with fine-grained environment.

*Coincident Variants*. For Baldwin, these were mutations that altered the phenotype in the same direction as the environmental modification, resulting in the eventual loss of plasticity.

*Correlated Variants*. For Baldwin, these were mutations that arose that improved the performance of other phenotypes that were permitted to survive by plasticity.

*Cryptic Genetic Variation*. Genetic variation that can accumulate in a population without being phenotypically expressed in normal environments, but which are expressed in novel environments.

*Ecological Speciation*. The production of new species through divergent selection in divergent environments, producing reproductive isolation as a byproduct.

*Evolutionary Rescue*. Genetic changes that occur when a population is on the brink of extinction, which improve fitness and prevent extinction.

*Flexible Stem*. This term is used to describe situations in which two or more, relatively non-plastic, sister species reside in distinct environments and are phenotypically distinct, while the ancestral population is plastic in such a way that at least some of this variation is captured by raising the ancestral population in either derived environment. That is, plasticity in the ancestor led to genetically based phenotypic diversification among daughter species.

*Genetic Assimilation*. Modern definition: adaptive loss of plasticity. Historical definition: it refers to the evolution of a reaction norm such that a phenotype not produced in the ancestral environment undergoes selection upon expression in a new environment, such that it can now be produced in the ancestral environment. This could involve the loss of plasticity, or shifts in reaction norm height and slope.

*Genetic Compensation*. The evolutionary rescue of populations experiencing maladaptive plasticity, in a manner that restores the phenotype from its modified state.

*Organicists*. Name given to an eclectic mix of biologists who argued for an important role for the environment in shaping the phenotype and directing the future course of evolution. This group included James Mark Baldwin, Henry Fairfield Osborn, C. Lloyd Morgan, and Edward Bagnall Poulton, among others.

*Organic Selection*. Baldwin’s term for the survival of plastic individuals and the course of evolution thereby taken.

*Orthoplasy*. Baldwin’s term for the direction of evolution determined by plasticity.

*Phenotypic Accommodation*. The process whereby genetic and plastic changes to an organism that disrupt its function are compensated by plastic changes in other phenotypes.

*Phenotypic Plasticity*. The ability of a single genotype to produce multiple environmentally induced phenotypes.

*Plastic Compensation*. The plastic equivalent of genetic compensation, and a subset of phenotypic accommodation. The plastic rescue of individuals experiencing maladaptive plasticity, in a manner that restores the phenotype from its modified state.

*Plasticity-Mediated Persistence*. The protection from extinction afforded by plasticity. Divided into hard PMP, where plasticity is essential for survival, and soft PMP, where plasticity is of adaptive significance in the new environment.

*Reaction Norm*. A function describing all of the phenotypic states possible for a genotype across some environmental variable.

*Standing Genetic Variation*. Genetic variation for a phenotype that exists in natural populations.

### B. Baldwin’s Theory of Speciation through Plasticity

Although Baldwin did not have modern theories of speciation to rely on, he did briefly discuss his own theory of speciation involving plasticity. The following theory has been taken largely from chapters 13 and 14 of Baldwin [6].

(a) A population of organisms that have the capacity to learn is geographically split into two identical populations.
(b) Both populations encounter the same novel environment.
(c) There are multiple ways to overcome the challenges imposed by this new environment.
(d) Individuals, through exploration and trial-and-error, discover a successful way to survive.
(e) Through social learning/imitation, this method is passed on to other members of the population and across generations.
(f) By chance, the strategy discovered by one population is different from the strategy discovered by another population.
(g) Coincident variation occurs to assimilate this strategy (making it instinctive to reduce the lag time involved with imitation), while correlated variation improves other aspects of the phenotype associated with this strategy. In this manner the two populations diverge behaviourally, morphologically, physiologically, and genetically.
(h) Although Baldwin had no theory of reproductive isolation, we could round his theory off by concluding that selection on these associated traits produces isolation as a byproduct. Hybrids may be inferior to either form, for instance.

Baldwin’s theory is therefore akin to the modern concept of ecological speciation, with the added twist that the populations do not encounter different environments, but rather create their own environments through behavioural plasticity within initially identical environments.

### C. Historic versus Modern Definitions of the Baldwin Effect and Genetic Assimilation

Baldwin and Waddington were not always clear writers. They often coined terms that were not accepted by the scientific community. Baldwin’s knowledge of inheritance was pre-Mendelian, and the mechanisms by which the Baldwin effect acts are often vague. It is no surprise then that misunderstandings over their ideas have arisen. One such misunderstanding is worth briefly discussing, as the paper it is found in, published in the journal* Evolution*, has become the definitive statement on the subject. Crispo [107] explicitly differentiates between the Baldwin effect and Waddington’s genetic assimilation by attributing to Baldwin the evolution of reaction norm elevation or* increased *plasticity, and to Waddington the adaptive* loss* of plasticity. She makes this error by misreading one particular passage in Baldwin [6] and misinterpreting one experiment of Waddington’s. The Baldwin passage, with Crispo’s interpretation in brackets, is as follows:
*many functions may be passably performed through accommodation, supplementing congenital (heritable) characters, which would be better performed were the congenital characters strengthened (i.e., if further phenotypic change occurred). Congenital variation would in these cases by seizing upon this additional utility (plasticity), carry evolution on farther than it had gone before (i.e., result in heritable changes in form)… this would give the gradual shifting of the congenital mean toward the full endowment (phenotypic optimum) ([6], pp. 209-210).*

The problem arises from Crispo’s interpretation of this passage: “An empirical example of shifts in mean phenotypic value without a change in level of plasticity is documented…” Crispo seems to interpret Baldwin as stating that plasticity of a trait can remain unchanged while the trait mean in either environment evolves in parallel, producing a change in reaction norm height but not slope (although in other places she includes an increase in slope to be another aspect of the Baldwin effect). Had she fully quoted Baldwin’s statement, Baldwin’s meaning would have become clear. As Baldwin writes:
*congenital variation would in these cases by seizing upon this additional utility carry evolution farther than it had gone before. For example, muscular strength in biting would in no way prevent the evolution of hardness of teeth. The accommodation factor (muscular strength) would be gradually dispensed with, since the most unsuccessful of those which depended upon accommodation would be eliminated. (brackets mine)*.**

In other words, Baldwin is* not* talking about trait means versus plasticity, as Crispo wants him to, but is rather talking about the evolution of nonplastic, correlated traits (hardness of teeth)* and *the loss of plasticity (muscular strength). In this example, Baldwin is suggesting that plasticity in trait A enables population persistence long enough for nonplastic trait B to evolve a function that supplants trait A. Plasticity in trait A had been costly due to the time required for trait A to respond to the environment; the result was its replacement by trait B and, potentially, the loss of the now-unecessary plasticity of trait A. Crispo’s interpretation causes her to define the Baldwin effect as follows:
*in the Baldwin effect, selection may act to change mean trait values without changing the level of plasticity in the population, or, alternately, selection acting on the phenotype can result in increases in the level of plasticity (because the most plastic individuals possess the most extreme phenotype and are thus positively selected)…Genetic assimilation, conversely, should act to decrease plasticity (i.e., increase canalization) in a population within a given range of environmental conditions if an increase in canalization is adaptive.*

On my reading this is an incorrect definition of the historical use of this term. The Baldwin effect was always interpreted to include the loss of plasticity as one form of orthoplasy [8]. Genetic assimilation similarly does not conform to Crispo’s definition.

Crispo quotes Waddington’s definition of genetic assimilation as a process “by which a phenotypic character, which initially is produced only in response to some environmental influence, becomes, through a process of selection, taken over by the genotype, so that it is formed even in the absence of the environmental influence which had at first been necessary ([108] quoted in [107]).” She then interprets this to mean that “the environment induces phenotypes that are adaptive, and then selection on the developmental system acts to reduce responsiveness to the environment (i.e., reduced plasticity), so that the induced phenotype becomes “inherited” (i.e., canalized) after a number of generations of exposure to the environmental stimulus [107].” This interpretation, in which a novel phenotype is plastically produced and then “assimilated” into the genotype as an adaptively nonplastic (or less plastic) trait, is the current prevailing definition of genetic assimilation, but it is not Waddington’s definition. First, Waddington defined canalization, not as the loss of plasticity, but as the buffering of the phenotype from environmental or genetic perturbations, and this could importantly include plastic phenotypes [9, 109, 110]. If a reaction norm that responded to temperature maintained its shape against novel mutations or nontemperature environmental factors, that plastic reaction norm would be canalized under Waddington’s definition. If one phenotype along a reaction norm resisted environmental or genetic perturbations, but a phenotype at the other end of the reaction norm revealed increased phenotypic variation under the same perturbations, the first phenotype but not the second would be canalized under Waddington’s definition. Thus canalization is broader than genetic assimilation, and plasticity itself can be canalized. Second, Waddington’s definition of genetic assimilation nowhere requires the loss of plasticity. So long as a phenotype that could not be produced in the old environment undergoes selection in the new environment such that it is now produced in the old environment, genetic assimilation has occurred. This is most readily understood through the loss of plasticity (which is a form of increased canalization) but could just as easily occur with a shift in the height or increase in the slope of plasticity. This is exactly how Waddington defines it in his 1959 paper [111] which Crispo believed Waddington to have misinterpreted. Here Waddington imposed selection on fruit flies for a phenotype induced by high salt concentration (large number of anal papillae) that was also associated with survival. After several generations a large number of anal papillae could now develop under low salt concentrations. This is genetic assimilation. It also happens that this selection resulted in increased plasticity of the phenotype—even more anal papillae were induced by high salt concentrations in these selected lines—so there were both an increase in the height (resulting in genetic assimilation)* and* an increase in the slope of the reaction norm. This is completely consistent with Waddington’s defintion of genetic assimilation and shows that he did not take canalization and genetic assimilation to be equivalent.

Ultimately, terms will be defined based on modern usage and their usefulness in describing natural phenomena. The Baldwin effect, genetic assimilation, and canalization have been defined in numerous review papers following Crispo’s definition, including some of my own (e.g., [93])—but it is at least worth noting that this is removed from how the generators of these terms originally used them, and too narrow of definitions may prevent the generation of new hypotheses. For instance, the idea that plasticity can be selected to be canalized against other environmental factors has received little attention.

### D. Learning and Invasive Success

Although Baldwin [6] allowed for all forms of plasticity (morphological, physiological, behavioural), learning was of especial interest to him. Learning has been called “hypervariable” plasticity [112] because it can result in the production of numerous behavioural (and in conjunction morphological, physiological) phenotypes in response to a diverse array of novel conditions. A particularly rich literature has developed around the importance of learning in the successful colonization of new habitats. Of especial interest are the dual roles of innovation through exploratory behaviour, and social learning through copying [113]. Organisms that exhibit exploratory behaviour are more likely to hit upon novel adaptive behaviours for surviving in novel environments; organisms that have strong social learning are more likely to mimic and retain adaptive behaviours. A combination of the two can permit innovation while reducing the risks of unnecessary exploration, what is known as the adaptive flexibility hypothesis [113]. For instance, invading sparrows showed greater innovation through their willingness to approach and consume novel foods than did long-established noninvasive sparrows of the same species [114]. Learning enables colonization of urban environments [77] and tends to be higher in invasive species than the conspecific species they displace [115–117]. Invasive species tend to show more innovative behaviour in their home range than noninvasive species [118, 119]. Brain size can act as a proxy for learning capacity, and has been shown to be positively associated with survival and invasive success [120–122], increased innovation [123], increased tolerance of environmental uncertainty [124], and reduced mortality rates [125]. For instance, a comparison of 446 introduction attempts in mammals found that successful colonization was significantly associated with brain size, even after accounting for life-history, body size, mating strategy, habitat generalism, and the number of released individuals [121]. Experimental evidence also supports PMP. Blue tits synchronize their breeding time with peak food availability by learning from the previous year’s environmental cues. Birds fed by researchers while they were nestling laid eggs later the following year, missing the peak production of their natural food. Control birds that relied on natural food showed no such delay the following year [126]. Thus plasticity to the wrong cues can be maladaptive, but a lack of responsiveness could be similarly maladaptive [113, 127]. Over one hundred years after Baldwin, evidence is rapidly accumulating that support his initial hypothesis that learning in particular is an important contributor to PMP.
